# Lipid biomarkers reveal dominance of aerobic methanotrophy in a continental serpentinizing system

**DOI:** 10.3389/fmicb.2025.1694997

**Published:** 2026-03-05

**Authors:** Cybele R. Collins, Mary N. Parenteau, Linda L. Jahnke, Michael D. Kubo, Serena Moseman-Valtierra, Kyle Young, Dawn Cardace

**Affiliations:** 1 Department of Geosciences, University of Rhode Island, Kingston, RI, United States; 2 NASA Ames Research Center, Exobiology Branch, Moffett Field, CA, United States; 3 Blue Marble Space, Seattle, WA, United States; 4 Department of Biological Sciences, University of Rhode Island, Kingston, RI, United States

**Keywords:** biomarker, groundwater, lipid, methanotroph, ophiolite, serpentinization, subsurface

## Abstract

Sources and sinks of methane within an advanced serpentinization-influenced system were investigated at the Coast Range Ophiolite Microbial Observatory (CROMO) in Lower Lake, California. Subsurface water-rock reactions at CROMO contribute to unique, high pH groundwaters and substantial methane emissions. We performed lipid analysis on biomass and measured radiocarbon and stable carbon isotopic composition of groundwater to trace the origins and fate of methane. Specific groups of microorganisms involved in methane cycling were identified through analysis of membrane lipid components. Aerobic methanotrophs dominated the samples, with evidence of heterotrophic bacteria but no detection of anaerobic methanotrophy or methanogens. Following these data, microbial activity may be a significant sink but not a major source of methane at this site.

## 1 Introduction

Subsurface rock-hosted water contains a wide range of microbial ecosystems that contribute to global biodiversity (Magnabosco et al., 2018a) and carbon cycling (Amend and Shock, 2001). Ultramafic-hosted hydrothermal vents, as found in the Lost City hydrothermal field (Kelley et al., 2005), share features and ecosystem dynamics with continental groundwater and spring-fed ophiolite systems (Blank et al., 2009; Cardace et al., 2013; Schrenk et al., 2013; Templeton et al., 2021). At these sites, reactions of ultramafic rocks and water produce serpentine-dominated mineral assemblages and extremely high pH waters, with hydrogen, methane and associated aqueous compounds available for consumption by microorganisms (McCollom and Shock, 1997; Schulte et al., 2006). Important limitations to growth include alkaline conditions (Etiope and Sherwood Lollar, 2013), a factor in the low inorganic carbon resources at these sites (Schrenk et al., 2013; Woycheese et al., 2015), and low oxygen (Mailloux and Fuller, 2003; Lang and Brazelton, 2020). The challenge of assessing biotic carbon cycling in these underdocumented subsurface environments calls for targeted and specialized strategies.

The unique geochemical environments hosted by serpentinizing minerals may have contributed to early life on Earth (Sleep et al., 2004) and may create similar conditions in planetary bodies within the habitable zone of a parent star (Russell et al., 2010). Serpentinizing systems are also of import to carbon cycling over deep time and contribute to abiotic carbon sequestration (Cipolli et al., 2004; Kelemen et al., 2011). However, total carbon sequestration in these environments, through mineralization of carbon dioxide, is countered by redox conditions that may drive high methane output (Etiope and Sherwood Lollar, 2013). The source and fate of methane in serpentinization-based systems, whether into biomass, rock, or the atmosphere, is insufficiently understood, though relevant to the application of methane as a biosignature in extraterrestrial settings. Methane from these sites may be geogenic, originating from the reduction of carbon in host rock or aqueous systems, and may also be biogenic, generated from, or consumed by, microorganisms (Brazelton et al., 2017; Schwander et al., 2023), which we targeted in our study.

Biotic methane cycling relies on methanogens and methanotrophs. Taxonomically, methanogens are Archaea, as are many anaerobic methanotrophs (ANME) (Hoehler et al., 1994). Other anaerobic methanotrophs include nitrite-reducing bacteria in the NC10 phylum (Ettwig et al., 2010). Among aerobic bacteria, methanotrophy is well documented in Gamma- and Alphaproteobacteria orders, though other taxa are active as well (Bodelier et al., 2009; Howe et al., 2023); for example, the metabolic diversity of verrucomicrobial methanotrophs, first discovered in acidic sites, is a topic of ongoing study (Schmitz et al., 2021). Aerobic proteobacterial methanotrophs use oxygen as a terminal electron acceptor via different metabolic strategies for carbon fixation that classify them as Type I and Type II (Nichols et al., 1985).

In serpentinization-influenced water-rock systems, methane cycling has been scrutinized using gene-based techniques. ANME have been found in seabed sites (Orsi et al., 2020) and the Samail Ophiolite (Howells et al., 2025). Type I (Gammaproteobacterial) methanotrophs have been detected in serpentinizing environments of the Lost City and Old City hydrothermal vent systems (Brazelton et al., 2006; Lecoeuvre et al., 2021), in New Caledonia (Quéméneur et al., 2023) and the Voltri Massif (Brazelton et al., 2017). Both Types I and II were also found in the Chimaera seeps (Rattray et al., 2022) and the Samail Ophiolite (Kraus et al., 2021). At CROMO wells, there are significant indicators of Type I gammaproteobacterial methanotrophy, including a full metagenome-assembled genome (MAG), and suggestions of Type II alphaproteobacterial methanotrophy (Seyler et al., 2020).

Methanotrophs offset biotic methanogenesis (Guerrero-Cruz et al., 2021). Estimates of methane emissions from aqueous systems and the methanotrophic effect on these emissions are still not well constrained despite extensive work, but have been calculated in seawater (Reeburgh, 2007), lakes (Gafni et al., 2024) and wetlands (Yang et al., 2025), while methanotrophy in soil ecosystems has already been identified as a significant influence on global methane emissions (Saunois et al., 2025). In shallow marine coastal waters alone, aerobic methanotrophs may uptake half of the available dissolved methane (Mao et al., 2022), comparable to methane consumption by the world’s forests (Pan et al., 2024). Recently, aerobic methanotrophs at locations previously assumed to be hypoxic or anoxic have been documented across lakes and other waterbodies (Berg et al., 2022; Schorn et al., 2024; Ruff et al., 2024). Here we assess the potential impact of methanotrophs in a low-oxygen, high-pH groundwater system.

At the Coast Range Ophiolite Microbial Observatory (CROMO) in the McLaughlin Natural Reserve, Lower Lake, CA, USA, scientific monitoring wells have been installed in serpentinite units to enable close study of subsurface waters. Previous work at CROMO and McLaughlin Natural Reserve wells tied to past mining activity on site found genetic evidence of methane-cycling microorganisms (Crespo-Medina et al., 2014; Twing et al., 2017; Seyler et al., 2020; Twing et al., 2025). We applied a targeted, lipid-based approach to distinguish between specific groups of microorganisms based on membrane composition. Forms and concentrations of specific lipids can be used to distinguish between living and dead cells and to assess microbial responses to changeable or extreme environmental conditions (Hazel and Williams, 1990; Costello et al., 2002; Parenteau et al., 2014; Rempfert et al., 2023). Fatty acids predominate in bacterial membranes, while archaeol is common in archaeal membranes (Nichols et al., 1985; Bowman et al., 1991; Jahnke et al., 1999; Bodelier et al., 2009). In bacteria, a unique sequential pattern of at least three monounsaturated fatty acid isomers can define Type I and Type II proteobacterial methanotrophs (Jahnke and Diggs, 1989), which have distinct cellular membrane features in contrast to other bacterial phylotypes.

Here, we use membrane-based lipid analysis and isotopic profiles of carbon to strengthen the case for active methanotrophy at CROMO. This work shows how methane supports low biomass or emerging microbial ecosystems and considers the fate of serpentinization-related methane: is it sequestered into biomass or lost to the atmosphere?

## 2 Methods

### 2.1 Site location in the Coast Range Ophiolite (CRO) and CROMO waters

**Figure 1:**
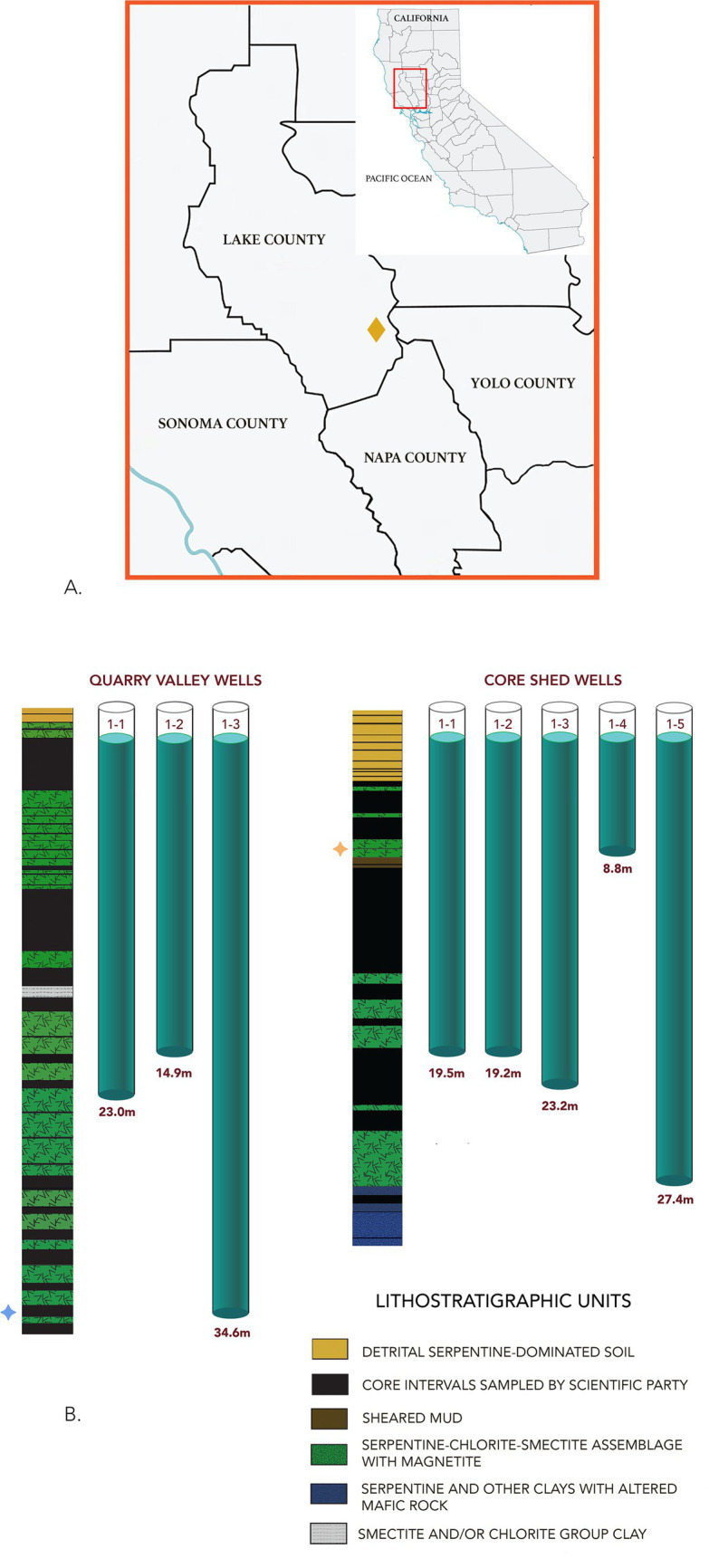
**(A)** Location of CROMO at McLaughlin Natural Reserve near Lower Lake, CA. **(B)** Schematic diagram showing the relative depths of the CROMO well array, Quarry Valley and Core Shed Wells, with reference to the lithostratigraphic levels from CSW1-1 cores. Depth of the well, in meters, is shown below each well in the cartoon. In the field, water levels differed slightly. Stars suggest zones of inferred lateral flow of water from formation in CSW1-4 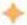 and QV1-3 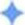. Redrawn after Cardace et al. (2013).

At the McLaughlin Natural Reserve locality of the CRO, ultramafics are exposed at the ground surface or overlain by thin soils with a shallow water table, facilitating groundwater access. The CRO units are tectonically blended in a classic mélange terrain. Variably metamorphosed ultramafic and mafic rocks exist in a structurally complex relationship with bounding sedimentary formations (Melosh et al., 2024), within a Jurassic age (172–161 mya) section of oceanic lithosphere (Choi et al., 2008). The CRO itself has been dissected and reworked by multiple collisional tectonic episodes followed by active faulting, resulting in a set of cogenetic (variably serpentinized) ultramafic blocks. Their modern locations, spaced geographically from a southernmost outcrop near Point Sal, CA, to points north of Stonyford, CA, are further impacted by action along the San Andreas and San Nacimiento Faults (McLaughlin et al., 1988; Shervais et al., 2004; Choi et al., 2008) (Figure 1A). Cores retrieved during CROMO drilling documented serpentine-rich soils to ~4 m depth and altered ultramafics containing serpentine group minerals and magnetite, as well as altered mafic materials dominated by chlorite, quartz, and calcite with some albite, suggesting both submarine and subaerial alteration (Cardace et al., 2013) (Figure 1B). Subsurface microbial communities in groundwater can be sampled from monitoring wells at CROMO that access mineralogical/hydrological conditions within two sites in a structural valley, less than 1.5 km apart: Quarry Valley (QV, to the west) and Core Shed Well (CSW, to the east) sites.

Waters observed in or near ultramafic rock outcrops such as those at CROMO include open system waters, with Mg^2+^ and HCO_3_^−^ as the dominant ions, produced through CO_2(aq)_ and O_2(aq)_-charged surface water infiltration through ultramafic country rock. Contrasting closed-system waters, dominated by Ca^2+^ and OH^−^, develop in subsurface reaction zones that are isolated from the atmosphere. An extremely elevated pH results, due to the hydroxide ion abundance (Barnes et al., 1967). The shallowest well, CSW1-4 (near historic ‘Core Shed’ location), accesses open system water with laterally advecting Mg^2+^-HCO_3_^−^ type waters that communicate with the atmosphere along the flow path. The deepest well, QV1-3 (Quarry Valley location) is fed by a different lateral flow regime through an essentially closed system, capped with a quasi-impermeable clay layer/aquitard (Cardace et al., 2013) in which high pH, Ca^2+^-OH^−^ type waters evolve (Barnes et al., 1967; Neal and Stanger, 1985). Deeply sourced CROMO wells (e.g., CSW1-1 and QV1-3) are typified by elevated pH and high methane. Near-surface, soil-impacted waters (e.g., CSW1-4) have near-neutral pH and relatively lower methane.

### 2.2 Lipid sample collection

Industry standard low-flow bladder pumps allow water sample delivery from well head ports at all CROMO wells. CSW1-4 (8.8 m) and QV1-3 (34.6 m) are cased with PVC to the bottom of each well, with a perforated interval at the bottom of each PVC installation through which waters enter the well at a particular depth.

In December of 2023 (rainy season) and May of 2024 (start of the dry season), we pumped the groundwater out of CSW1-4 and QV1-3 to sample for lipid content. The water column was drawn from the bottom of each sampled well with positive displacement Teflon bladder pumps,^1^ then run through an airtight flow cell holding a YSI 556 multiprobe meter documenting temperature, pH, ORP, dissolved oxygen (May 2024) and conductivity measurements in real time. We conducted a field purge of the wells of interest, pumping the standing water out of wells, then allowing the wells to recharge over a period of 5–6 days.

To capture intact and fragmented microbial cells and then analyze constituent lipids, we performed a vacuum filtration of water at the well head, discharged through well exit-tubing into a baked ceramic holder containing a 0.7 μm glass fiber filter (GFF). GFFs were baked at 450 °C for 8 h prior to use to remove all traces of organic material and stored in baked foil wrappers until use. In the field, we pumped a range of 63–656 mL of water through the GFF until biomass and mineral content clogged the filter. We removed the GFF using baked metal forceps and placed it into aluminum foil baked at 550 °C for 12 h, gently folded the packet and immediately placed it on dry ice. Packets were transported to NASA Ames Research Center in Moffett Field, CA on dry ice, then frozen at −80 °C until analysis. In addition, a carboy of water was collected in December of 2023, kept at 4 °C for approximately 4 h and filtered at the field station, with treatment of the GFF as above. Biofilm collected with GFF swabs was obtained from a slender PVC pipe submerged in CSW1-1 for more than 24 months. Lipid content from pre-purge and post-purge waters were collected for comparison for QV1-3 and CSW1-4.

### 2.3 Lipid extraction, derivatization and analysis

Total lipids (TL) were extracted from CROMO GFFs and biological controls using a modified Bligh-Dyer (BD) extraction procedure (Jahnke et al., 1992) and two fractions prepared sequentially from the TL extracts (Bligh and Dyer, 1959). First, TL were treated using a mild alkaline methanolysis (MAM) to recover fatty acyl moieties from polar membrane phospholipid and glycolipid as methyl esters (FAME). The lipid residue from MAM was recovered by an additional BD extraction and treated using an acid methanolysis procedure to recover free fatty acids (FFA) and alkyl and phytanyl glycerol ethers with further derivation with trimethylsilyl (TMS) (Jahnke et al., 2001). The double bond positions of FAME were determined as dimethyl-disulfide (DMDS) prepared using a sensitive procedure described by Yamamoto et al. (1991) with the addition of two internal standards, C_20:1∆11_ and C_22:0∆13_ (see Table 1 footnote). Samples were analyzed as FAME or TMS derivatives using an Agilent 5977A Gas Chromatograph–Mass Selective Detector (GC–MSD) equipped with a 60 m DB5ms fused silica column. Chromatographic conditions for FAMEs (280 °C injector) were 50° to 170°C at 10°C/min, 170° to 260° at 1°C/min, 260°to 320° at 10°C/min, held isothermally for 5 min. Procedural blanks were run. Conditions for DMDS adducts were the same, but the isothermal phase was held for 130 min for recovery of all DMDS-adducts. All fractions containing FAME, FFA or glycerol ether moieties [monoalkyl, dialkyl, or diphytanyl (MAGE, DAGE or archaeol, respectively)] were identified through retention times and characteristic mass-to-ion charge ratio (m/z) fractionation patterns of their molecules relative to controls and available standards.

**Table 1:** Biological and chemical lipid standards used to help interpret the environmental lipid data.

Microbes and chemical standards	Metabolism: aerobic or anaerobic oxidation of methane (AOM)	Distinguishing lipids	References
*Halobacterium* sp.	AOM: proxy for ANME	Archaeol	Tomlinson et al. (1986) and Sprott et al. (1997)
*Sulfolobus* sp.	AOM: proxy for ANME	Archaeol	Sprott et al. (1997) and Trent et al. (2003)
*Thermocrinis ruber*	AOM: proxy for SRB	MAGE/DAGE	Nichols et al. (1993) and Jahnke et al. (2001)
*Methylococcus capsulatus*	Aerobic: Type I methanotroph	C_14:0_, C_16:1Δ9_, C_16:1Δ10_ C_16:1Δ11c_, C_16:1Δ11t_, C_cyc17:0_	Makula (1978) and Jahnke and Nichols (1986)
*Methylosinus trichosporium*	Aerobic: Type II methanotroph	C_18:1Δ10c_, C_18:1Δ10t_, C_18:1Δ11t_ and C_18:1Δ11c_*	Makula (1978) and Nichols et al. (1985)
FAME standard mix	N/A	C_14:0_, C_14:1_, C_16:1∆9c/t_, C_16:0_, C_17:1_, C_18:1Δ9_, C_18:1Δ11_, C_19:1_, C_19:0_, C_20:1_, C_20:0_, C_21:0_, C_22:1_, C_22:0_	Created by LL Jahnke for this study

Fatty acid nomenclature: e.g. C_18:1∆9c_ indicates an 18 carbon chain, with one double bond at the ninth carbon (∆9) from the carboxyl end of the molecule, with c (*cis*) configuration, as opposed to t (*trans*).

*C_18:1Δ10c_ was abundant but C_18:1Δ9_ was not found in this *Methylosinus trichosporium*. Both are present in other Type IIs (Bowman et al., 1991).

The microbes were chosen because they contain key lipids that would act as proxies for detecting evidence of either aerobic or anaerobic oxidation of methane: archaeol (universal biomarker for Archaea), monoalkyl glycerol monoether (MAGE) and dialkyl glycerol diether (DAGE), and fatty acids diagnostic for the aerobic oxidation of methane by Type I and Type II methanotrophs. Other lipids target anaerobic oxidation of methane (AOM) consortia of anaerobic methanogenic archaea (ANME) and sulfate-reducing bacteria (SRB).

In total, a set of 33 lipid samples representing these GFF filters were analyzed from seven wells: CSW1-1, CSW1-2, CSW1-3, CSW1-4, CSW1-5, QV1-2 and QV1-3. Of these, 32 samples showed at least one bacterial fatty acid peak (Supplementary Table ST1). Twenty samples were analyzed for fatty acids. In addition, we prepared two types of controls (see Table 1): a chemical control, composed of FAME lipid standards common among bacteria, and a biological control, using biomass from microbial cultures previously grown in the NASA ARC laboratory. The controls contain lipids representative of the major metabolic types of aerobic and anaerobic methanotrophy, which were combined into a quality assurance sample to represent possible lipid types of methanotrophs, SRBs, ANME, methanogens and other microbes. In the biological control were Gammaproteobacterial *Methylococcus capsulatus* Bath, a Type I methanotroph, and an Alphaproteobacterial *Methylosinus trichosporium*, a Type II methanotroph. For lipids found in archaea and syntrophic SRBs of ANME, we used *Halobacterium* sp., a halophile that produces large amounts of archaeol; *Sulfolobus* sp. for archaeol and GDGTs; and an Aquificales sp. (*Thermocrinis ruber*) as a source of DAGE, identified in the ANME-SRBs consortia (Blumenberg et al., 2004).

We initially analyzed filters prepared for FAME, which represents the ester-bound fatty acids associated with intact membrane polar lipids. These polar lipids are rapidly degraded to free fatty acids when a bacterial cell dies and thereby may characterize living cells when captured by conversion to FAME (White et al., 1993). The cytoplasmic membrane of all bacteria is composed of roughly similar amounts of phospholipid fatty acids (PFLA) and can be extrapolated to cell numbers (White et al., 1993; Jahnke et al., 2008), dependent on relative cell size. We estimated cell count for a filter with the most robust biomass and clear methanotrophic signals (QV1-3 f3). White et al. (1993) suggest that a value of 2.5 × 10^10^ cells μmol applies to subsurface bacteria and that 200 nmol FA/mg to represent bacterial carbon, of which approximately 50% are fatty acids. We used an internal standard, 0.5 ng of C_23:0_, adjusted to peak level for cell count calculations in QV1-3 samples. Nanomolar conversions were used with estimations above. Through White’s calculations, we converted to 9.8 × 10^3^ fg carbon on the filter, using 3.76 × 10^2^ fg bacteria based on a 26 fg/cell carbon (Trousselier et al., 1997) and divided by the amount filtered (137 mL) for cell count concentration.

### 2.4 Methane emissions

Optical feedback-cavity enhanced absorption spectroscopy (OF-CEAS) provided near-instantaneous concentration data for methane in headspaces of wells, using the LI-COR 7810 field portable trace gas analyzer (LI-COR Environmental, Lincoln, NE 68504).^2^ Gas emissions at well heads were measured by connecting the LI-COR in a closed loop via tubing (Bev-A-Line) to a customized PVC cap (2-inch-diameter) using gas-tight stainless-steel fittings. The cap was placed on top of each of the six 2-inch-diameter wells (CSW1-2, CSW1-3, CSW1-4, CSW1-5, QV1-2, and QV1-3). In December 2023, measurements of methane concentrations lasted for 4 to 6 min and were done in triplicate, with spacing of approximately 5 min between measurements, before and after purging wells. These measurements were made in conjunction with collection of YSI multiprobe data for environmental parameters. Calculations of flux followed Martin and Moseman-Valtierra (2015): All gas fluxes were calculated using the ideal gas law, from linear rates of change in gas concentrations in the headspace over time (Supplementary Figure SF2). These were used to model the outgassing from a particular well, rather than a standard measurement for the landscape, following Leong et al. (2023), and were reported as moles/yr from average flux rate in each study site by removing area of the well (0.008 m^2^).

### 2.5 ^14^C and δ^13^C sampling and analysis

In May 2024, samples from drained and refilled wells were collected for ^14^C radioisotope and δ^13^C stable isotope content of methane and dissolved inorganic carbon (DIC), according to procedures at the National Ocean Sciences Accelerator Mass Spectrometry Facility (NOSAMS).^3^ Two contrasting wells in depth and water access were sampled (CSW1-4 and QV1-3), as well as two wells that produced high methane emissions after recharge of water (CSW1-3 and CSW1-5). One of the latter (CSW1-3) had previous evidence of methanotrophy (Seyler et al., 2020). Samples were poisoned with HgCl_2_. DIC bottles were provided by the facility (1 L total). Methane samples were collected into pre-flushed septa-sealed serum vials (240 mL total), extracted on the vacuum line, then combusted to purify. For calibration purposes only, δ^13^C was measured by isotope ratio mass spectrometry (IRMS). The accelerator mass spectrometry (AMS) system at NOSAMS directly counted atoms of ^14^C using a modified MC-SNICS ion source and a 500 kV pelletron accelerator (National Electrostatics Corporation) with a bounced injection system. A primary standard of NBS Oxalic Acid I (NIST-SRM-4990) was used. Methane was treated as combusted organic carbon samples, with a blank of KHP (Sigma Aldrich) IAEA C-7 as a secondary combustion standard and ^14^C-free groundwater for DIC samples, along with in-house secondary standards. The blank for extracting the methane was checked immediately prior to measurement and was <0.2 μg C for the period of collection. Fraction modern (Fm) was reported, the deviation of the radiocarbon content of a sample from the modern standard [95% of the radiocarbon concentration (in AD 1950) of NBS Oxalic Acid I]. Samples and standard were normalized to a ^13^C_VPDB_ value of −25‰ by simultaneously measured ^13^C/^12^C ratios. Radiocarbon ages were calculated using the Libby half-life of 5,568 years (Stuiver and Polach, 1977). NOSAMS calculated an internal statistical error using the total number of ^14^C counts measured for each target and external error is calculated from the repeatability of multiple measurements of a given cathode over the course of a run, with the larger error reported. δ^13^C results as reported were measured on a split of sample carbon (CO_2_) by Thermo 253 Plus IRMS Dual Inlet/μVolume Analysis at Oregon State Stable Isotope Collaboratory (OSSIC), https://ceoas.oregonstate.edu/oregon-state-stable-isotope-collaboratory. The CO_2_ was analyzed by dual inlet isotope ratio mass spectrometry on a Thermo 253plus IRMS. OSSIC uses CO_2_ reference gas standards for daily runs that are calibrated to international standard NIST 8541 (NBS19).

### 2.6 Aqueous geochemistry

In January of 2025, concentrations of dissolved (total) K, Ca, Mg, Na, Zn, Cu, Mn, and Fe were measured in accordance with U.S. EPA Method 200.7 (1994) at the University of California at Davis Analytical Laboratory, https://anlab.ucdavis.edu/, with inductively coupled plasma-atomic emission spectrometry. Fluids were sampled in the field with well-rinsed tubing and passed through 0.22 μM PES filters (Millipore Sigma Sterivex Part No. SVGPL10RC); samples were kept at 4 °C prior to submission. Concentrations of ions Br^−^, Cl^−^, F^−^, NO_2_^−^, NO_3_^−^, PO_4_^3−^, and SO_4_^2−^ were measured by ion chromatography, in accordance with U.S. EPA Method 300.1 (1997), at the University of California at Davis Analytical Laboratory, https://anlab.ucdavis.edu/. Total HCO_3_^−^ and CO_3_^2−^ were measured according to Clesceri et al. (1998) at the University of California at Davis Analytical Laboratory, https://anlab.ucdavis.edu/. In June of 2025, complementary data for dissolved oxygen (separate from previous YSI measurements) were measured by FireSting GO_2_ sensor, according to manufacturer’s instructions, from PyroScience, Aachen, Germany, https://www.pyroscience.com/en/: the 5-m-long DO fiber optic cable (PN PS-OXROBGSC-CL5) was gently affixed to a foam-wrapped steel fish tape reel with continuous data collection as the cable was advanced in 2-cm increments into the well. Volatile fatty acids, including pyruvate, acetate and butyrate, were sampled by flushing a new, sterile sampling syringe 3 times with groundwater, expelling ~60 mL through a 0.22 μM PES filter (Millipore Sterivex, as above) to condition the filter prior to sampling into a 60 mL glass screw cap vial (Cole-Parmer Clear Precleaned EPA vials, PN 9953525) with polypropylene cap and ⅛ inch PTFE-faced silicone septum. Vials carry a certificate of analysis to meet EPA criteria for more than 60 volatile organic analyses (VOA). Samples were measured by HPLC equipped with a single wavelength UV detector at 210 nm, by Eurofins Orlando, Altamonte Springs, FL, https://www.eurofinsus.com/environment-testing/.

## 3 Results

### 3.1 Lipids document aerobic methanotrophy

**Figure 2:**
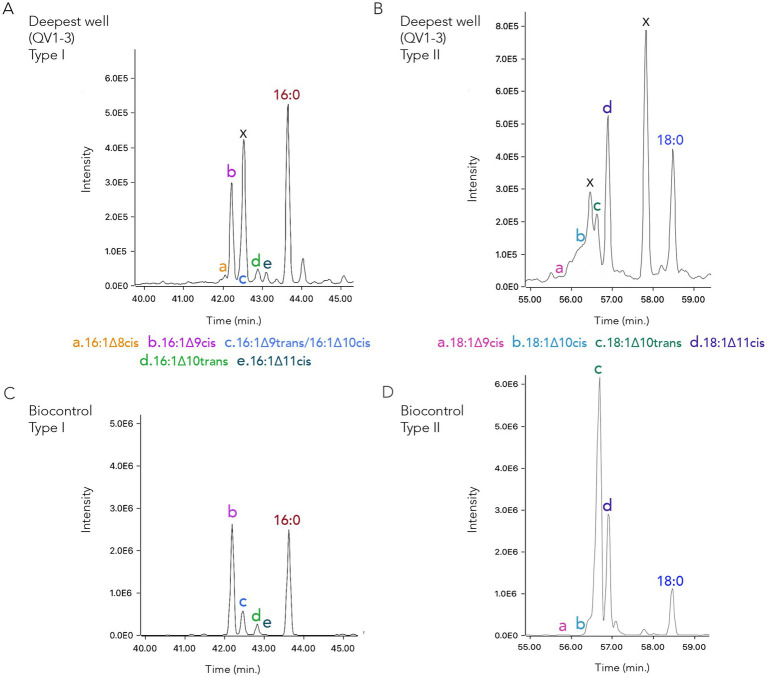
Biomass from the deeper CROMO well groundwater, QV1-3, indicates aerobic methanotrophy **(A,B)**. Peaks corresponding to retention times of specific molecules are shown from GC-MS analysis of fatty acid methyl esters (FAMEs), generated from mild alkaline methanolysis (MAM). The retention times in the biocontrol **(C,D)** of the monounsaturated fatty acids indicative of Type I and Type II methanotrophs were used to identify sample lipids. These data supported identification by the mass ion of the FAME and characteristic fractionation patterns, confirmed by DMDS. Above is one sample from well QV1-3 in two clusters of peaks **(A,B)**. The biocontrol **(C,D)** includes methanotrophic controls *Methylococcus capsulatus* Bath (Type I) and *Methylosinus trichosporium* (Type II) for reference to retention time. In identification of Type I methanotrophy **(A)**, six C_16:1_ monounsaturated peaks were identified via both methods. Type II methanotrophy **(B)** was identified by four C_18:1_ monounsaturated peaks. Please note that co-elution occurs with C_16:1∆9_ trans and C_16:1∆10_ cis, and that in **(A)** a monounsaturated peak corresponding to the co-elution - identified by fractionation pattern via ion extraction - is obscured by an unknown background signal in the QV1-3 well sample, marked by the X; another unknown signal is shown later **(B)** in the sample between the C_18:1∆10cis_ and C_18:1∆10trans_ peaks. Saturated C_16:0_ and C_18:0_ FAME are also indicated.

Aerobic methanotrophs of both major (RuMP and serine) metabolic pathways were identified through patterns of the fatty acids. In identification of Type I (RuMP) methanotrophy, a sequence of 16:1 (monounsaturated) peaks were identified by the characteristic fractionation pattern and the mass ion of peaks. They also corresponded to retention times in the *Methylococcus capsulatus* Bath control. Three C_16:1_ peaks as a sequence (∆8, ∆9, ∆10 or ∆9, ∆10, ∆11) are needed for positive identification of a Type I methanotroph. Including cis/trans isomers, we identified six C_16:1_ peaks (∆8c, ∆9c, ∆9 t, ∆10c, ∆10 t, ∆11c), confirmed by DMDS. Four C_18:1_ isomers were detected in our samples; three characterize a Type II (serine) methanotroph. Bond positions of isomers were first identified through relative retention positions (Nichols et al., 1985), confirmed by DMDS, which revealed ∆8 isomers in some controls and within one sample that corresponded to the retention time peak noted in DMDS samples (Figure 2). All analytical runs are described in Supplementary Table ST1, including lipid peaks for each of the 20 FAME samples. Samples were analyzed from the rainy and dry seasons (December and May, annotated as D/M below) and standing (pre-purge) vs. recharged (post-purge) water (S/R), with one sample filtered from water within 8 h of collection (CSW1-4_DR).

Following White et al.’s (1993) analysis of carbon on filter 3, we calculated 1.44 ng fatty acid, and found this filter trapped 1.38 ×10^8^ cells, or 1.01 × 10^6^ cells per mL (Supplementary Table ST2), which may be an upper limit on biomass as the signals were especially robust on this filter.

Every well we sampled indicated biomass in terms of C_16_ and C_18_ FAME, ubiquitous in membranes of bacterial cells. Specific markers were clear only in a subset of the samples. Background signals from samples obscured some FAME peaks that may include C_18:2_, a biomarker for Type II methanotrophs with two double bonds (Bodelier et al., 2009). Signals were present in relatively low concentrations in one filter from QV1-3 and CSW1-4, and in the CSW1-1 biofilm, but its levels were obscured with background signal (Supplementary Table ST1).

**Figure 3:**
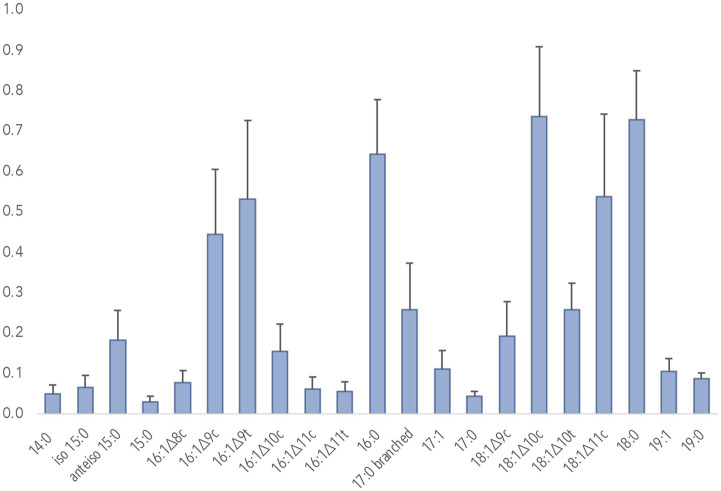
FAMEs observed in QV1-3 filters. Proportions shown are averaged data from five filters collected in sequence from one well and analyzed individually. C_20_, C_21_, and C_22_ saturated and monounsaturated fatty acids were also detected in trace amounts but obscured by background signals. Branched, *cis,* and *trans* isomers were determined by proportions to known elution patterns were confirmed on selected samples by DMDS.

There were no signs of archaeol on the derivatized MAH samples (Supplementary Figure SF1), which represented four wells. This lipid is common to both anaerobic methanotrophs and methanogens (Hinrichs et al., 2000). As with archaeol, DAGE and MAGE, found in sulfur-reducing bacteria, the syntrophs for ANME, were identified in controls but not located in samples. FAME signatures common to other ecologically pertinent bacterial groups (iso- and anteiso branched C_15_ and C_17_, cyclopropyl C_17_) were detected but at low levels within the mix of FAME chromatograms, as contrasted to proportions of signatures corresponding to methanotrophs (Figure 3). We also found background signals that we were unable to identify as they were not present in procedural blanks nor known to be common to drilling fluids (not used for CROMO but possible remnants in groundwater from earlier mining operations).

### 3.2 ^14^C radioisotope data and δ^13^C stable isotopes constrain fractions of old and modern carbon

δ^13^C was reported as −18.64‰ for dissolved inorganic carbon (DIC) and −31.05‰ for methane in the shallow well (CSW1-4), and −33.79‰ for DIC and −26.25‰ for methane in the deep well (QV1-3) (Table 2). From the same samples, radiocarbon in DIC was reported as about half of modern values (0.5073 ± 0.0015) in CSW1-4 waters, and nearly a third of modern values (0.3065 ± 0.003) in QV1-3 waters. In contrast, very little modern methane was discovered. Data reduction suggests ~4% of modern methane in CSW1-4 samples and only 0.08% of modern methane in QV1-3 samples (0.0008 ± 0.0016). Results from wells CSW1-3 and CSW1-5 are shown in Supplementary Table ST3.

**Table 2:** ^14^C radiocarbon and stable δ^13^C data for both dissolved methane and dissolved inorganic carbon (DIC), for samples collected after wells were purged then recharged, by atomic mass spectroscopy.

	**CSW1-4**	**QV1-3**
δ^13^C		
DIC (‰)	−18.64	−33.79
CH_4_ (‰)	−31.05	−26.25
^14^C		
DIC F Modern	0.5073 ± 0.0015	0.3065 ± 0.003
DIC Age	5,450 ± 25	9,500 ± 80
^14^C		
CH_4_ F Modern	0.0366 ± 0.0022	0.0008 ± 0.0016
CH_4_ Age	26,600 ± 490	>48,000

The shallowest well (CSW1-4) is marked in yellow and the deepest (QV1-3) in blue. For methane δ^13^C, average sample standard deviation per μl volume is 0.10‰. For DIC δ^13^C, average sample standard deviation (dual inlet analysis) is 0.02‰. Fraction modern reports the deviation of the radiocarbon content of a sample from the modern standard. In terms of ^14^C_DIC_, the DIC appears to be younger than methane. In terms of ^14^C_CH4_, the methane inventory is markedly older. Age units are years: 5000 to 10,000 yrs., Pliocene–Miocene; 26,600 yrs., late Oligocene; 48,000 yrs., mid-Eocene.

### 3.3 Aqueous geochemistry establishes different chemical environments across the CROMO well array

We monitored environmental parameters such as temperature, pH, conductivity for the sampling trips that included lipid collection in December 2023 and May 2024. We collected data on geochemical composition of fluids from 2023 to 2025 sampling trips and compared it to past data (Supplementary Table ST4). Samples for dissolved solute and ion measurements were collected in the rainy season (January 2025).

All data are presented in Table 3 to showcase the diversity of groundwater characteristics in the CROMO wells, which exert first order controls on microbial community composition. Well CSW1-1 is uncased below the soil-bedrock contact and thus is open to the surrounding rock formations, with ultrabasic (>11) pH. CSW1-4 sources waters that derive from serpentinized soil with oxidizing ORP. QV1-3 sources waters that derive from fractured ultramafic rocks amid clay with relatively reducing conditions. In January 2025, we collected post-purge geochemical data for major elements and important ions for these wells. Two replicate measurements were taken for wells CSW1-1 (pre-pump), CSW1-2 (post-pump) and QV1-3 (post-pump), while all others were taken in single measurements.

**Table 3:** Geochemical composition of fluids from 2023–2025 sampling trips.

	**CSW1-1**		**CSW1-4**		**QV1-3**	
	**Standing**	**Recharged**	**Standing**	**Recharged**	**Standing**	**Recharged**
Well depth (m)	19.5	19.5	8.8	8.8	34.6	34.6
pH	12.3*	12.2*	7.84	8.46	9.42	10.06
Temp. (C°)	15.2*	15.9*	16.88	15.35	17.2	17.84
ORP (mV)	−364.2*	−323.2*	310.8	102.46	−187.7	−256
SC (mS/cm)	3.317*	3.226*	2194*	2358*	4.054	5.977
DO (mg/L)	1.61*	1.29*	6.38*	1.94*	1.23*	1.35*
K (μM)	446.5	433.5	114.6	146.8	390.3	405.9
Ca (μM)	65	85	150	135	1030	905
Mg (μM)	BDL	0.02	630	580	BDL	BDL
Mn (μM)	BDL	BDL	BDL	0.31	BDL	BDL
Na (μM)	19.2	18.3	249.3	270.7	754.1	409.9
Fe (μM)	1.29	1.31	BDL	BDL	BDL	BDL
Br^−^ (μM)	4.7	4.88	12.7	12.6	40.17	24.2
Cl^−^ (μM)	5642	6827	15712	15740	55092	29140
F^−^ (μM)	3.95	2.63	BDL	BDL	BDL	BDL
NO_3_^−^ (μM)	BDL	BDL	97.3	33.5	19.5	BDL
SO_4_^2−^ (μM)	171.6	127.5	307	294.2	9.7	27.0
HCO_3_^−^ (μM)	BDL	BDL	2.4	2.8	BDL	BDL
CO_3_^2−^ (μM)	18.5	17.1	0.9	0.8	0.5	1.8
C_3_H_4_O_3_ (μM)	N/A	N/A	N/A	BDL	N/A	18.4

*ORP, SC and DO measurements taken with ion samples in Jan 2025, reported to compare pre- and post-purged water (standing/recharged). Other ORP, SC etc. were taken with lipid samples.

Measurements below are from December 2023; full sets for the wells during lipid sampling, including May 2024, are reported in Supplementary Data. Samples for ion levels were taken in Jan. 2025 in conjunction with DO and limited measurements of ORP and SC. Samples for organic acids were taken in June 2025 on CSW1-4 and QV1-3 only. Cu, Zn, NO_2_ and PO_4_ were below detection in all wells sampled. Well CSW1-1 (gray) is open to multiple stratigraphic layers; CSW1-4 (yellow) is open to serpentinized soil with oxidizing ORP; and QV1-3 (blue) is open to fractured ultramafic rocks amid clay with relatively reducing water.

**Figure 4:**
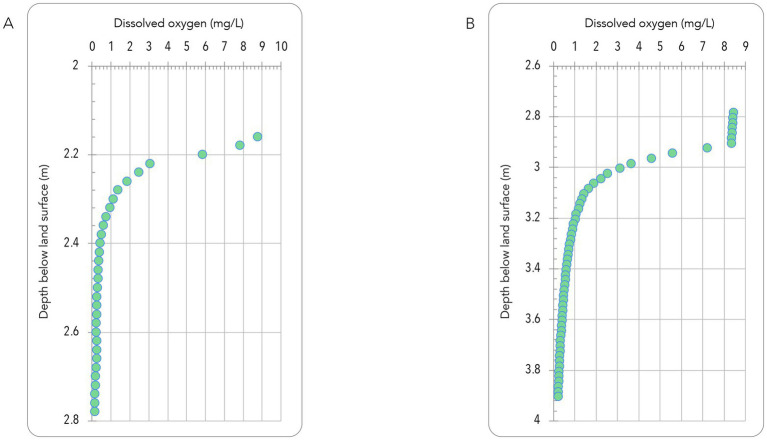
Dissolved oxygen data in mg/L demonstrates plummeting values to suboxic levels (<0.25 mg/L) within 2.2-4 m below ground surface for **(A)** standing water in CSW1-1 (condition of water for biofilm data) and **(B)** recharged water in QV1-3. These data suggest the depth beyond which atmospheric oxygen diffusing from above should not persist in abundance.

To document changes in dissolved oxygen (DO) levels below the water table in the wells, systematic observations were collected in a follow-up field campaign in June 2025. Suboxic DO was documented as less than 0.25 mg/L at 2.8 m below land surface at QV1-1 (uncased below soil horizon), less than 0.25 mg/L at 2.5 m below land surface at CSW1-1 (uncased below soil horizon), less than 0.25 mg/L at 3.8 m below land surface at QV1-3 (Figure 4).

Comparing dissolved concentrations of diverse analytes in pre- and post-purge well water samples reveals some differences between pre- and post- samples, and well-to-well differences. Total dissolved concentrations of K, Ca, Mg, Mn, Na, Fe (μM) are presented; ion concentrations of Br^−^, Cl^−^, F^−^, NO_3_^−^, SO_4_^2−^, HCO_3_^−^ and CO_3_^2−^ (μM) are presented (see Table 3).

Solute measurements between the recharged wells for K were within uncertainty for the method between two replicates taken for CSW1-1. K observations were 4% higher in post-purge samples for deeper QV1-3, and 28% higher in post-purge samples for shallower CSW1-4. Ca post-purge was 31% higher than pre-purge waters in CSW1-1 and 19% higher in QV1-3; post-purge water was 10% lower in Ca for CSW1-4. Mg was detectable in recharged CSW1-1 but below detection limit in standing waters. Mg was below detection in both conditions for QV1-3. Mg was 10% lower in the recharged waters for CSW1-4; CSW1-4 waters had more than 50-fold the Mg concentration of CSW1-1. Recharged Na was 7% less than standing water for CSW1-1, 1% higher for QV1-3 and 9% higher for CSW1-4.

Anion concentrations were dynamic. Recharged chloride was 20% higher for CSW1-1, the same for CSW1-4 and 8% higher for QV1-3. Recharged fluoride was 17% lower for CSW1-1 but BDL for all others. Recharged bromide levels for CSW1-1, CSW1-4 and QV1-3 were at a 0 to 2% difference to standing bromide (0.7 mg/L, 2.0 mg/L, 6.5 mg/L, respectively). Concentrations of polyatomic anions varied generally pre- and post-purge, and site-to-site. Nitrate was at highest levels in shallow CSW1-4 and in its pre-purge standing water sample. At this well, pre-purge water was three times as concentrated in nitrate as post-purge recharged water. At other wells, nitrate was low but present in standing water but BDL in recharged water (e.g., QV1-3). Sulfate varied between wells: recharged waters were 76% lower in CSW1-1, 4% lower in CSW1-4, and more than 2x higher in QV1-3.

Bicarbonate and carbonate ion abundances were determined separately from other ions. Levels were below detection for both HCO_3_^−^ and CO_3_^2−^ in CSW1-1 and QV1-3. CSW1-4 water was elevated by 14% with respect to HCO_3_^−^, but no CO_3_^2−^ was detected in recharged water.

Only CSW1-4 had detectable dissolved manganese (0.31 μM). Only CSW1-1 had detectable dissolved iron (~1 μM); recharged and standing water samples were the same for iron in CSW1-1.

Below detection were copper (<0.0 mg/L), zinc (<0.005 mg/L), nitrite (0.05 mg/L), phosphate (<0.05 mg/L) and acetate (<0.18 mg/L).

Recharged water from QV1-3 and CSW1-4 were sampled for volatile organic acids (June 2025). All were below detection limits except pyruvic acid, found in QV1-3 (1.6 mg/L, or 18.38 μM).

Fluids in all wells with a pH above 9 had relatively reducing oxidation–reduction potential on recharge (full data not shown). ORP results are reported in Table 3 (mV) prior to conversion to Eh to align with similar reported values (Seyler et al., 2020). CSW1-1 had Eh values ranging from −164.2 mV (standing) to −123.2 mV (recharged) and QV1-3 ranged from +12.3 mV to −256 mV. In contrast, CSW1-4, the well nearer to a neutral pH, had values of +510.8 mV for Eh (standing) and +302.46 mV for Eh (recharged).

### 3.4 Methane emissions in relation to lipid biomass

Methane emissions were detectable and pronounced from wells. Before and after purging wells in December 2023, and while sampling the recharged water, we measured methane emissions directly from six wells (QV1-2, QV1-3, CSW1-2, CSW1-3, CSW1-4, CSW1-5) and estimated flux in order to compare emissions in each 2″-diameter monitoring well (Supplementary Figure SF2).

Orders of magnitude differences were observed in methane concentrations before and after the groundwater recharge of wells of interest (Table 4). In the deepest well sampled, QV1-3, methane concentrations were first around 50x global averages of atmospheric, at 80–100 ppm vs. 1.9 ppm. After pumping out water, 5,000× atmospheric methane was measured (the day after pumping) at ~9,500 ppm, the highest detectable limit of the LI-COR. This lowered to 1,040–1,250 ppm or 600× atmospheric after 5 days of recharge, still more than an order of magnitude above initial levels. However, in the shallow well sampled, CSW1-4, methane values initially diminished from 20× atmospheric to 3× atmospheric (after 1 day), then approached atmospheric concentrations after refill (5 days).

**Table 4:** Headspace methane concentrations and outgassing of wells CSW1-4 and QV1-3 in December 2023 after pumping within 24 h and after recharge of fluids (5 days post-purge).

CSW1-4 and QV1-3	Lower range (ppm)	Upper range (ppm)	Outgassing (mol/yr)
Post-purge	5.3	5.6	2.5
Recharge	2	2.2	−0.1
Standing	34.8	55.1	3.4

QV1-3	Lower range (ppm)	Upper range (ppm)	Outgassing (mol/yr)
Post-purge	9,500	9,500	86134.3
Recharge	1,020	1,250	32.8
Standing	70	109	3.6

Standing water values, from 4 months post-pump, were measured before pumping and represent water with access to the atmosphere. Value range taken from variable slope of emissions. Note that the post-purge of QV1-3 was at the highest detectable limit (9,500 ppm). Outgassing per condition was estimated by multiplying a flux calculation by area of well opening.

Replicate measurement periods are reported as a range, as emission levels steadily rose through 5-minute sampling in most wells. Immediately after pumping or after recharge, 5 days later, emissions from all wells besides CSW1-4 either increased immediately after pumping or at recharge (Supplementary Figure 2). In order to compare to standard measurements of methane emissions (Etiope et al., 2016; Leong et al., 2023), we calculated methane flux for the area of the 2-inch-diameter pipes, which essentially create an artificial gas seep or point source of outgassing, comparable to bubbling sites (Leong et al., 2023); these do not represent a classic, natural landscape as in the chamber methods on which standard calculations are based (Martin and Moseman-Valtierra, 2015). Outgassing was then estimated per condition in the style of Leong et al. (2023) by multiplying flux with area.

## 4 Discussion

### 4.1 Evidence of aerobic methanotrophs in serpentinization-influenced systems

Aerobic methanotrophy appeared to dominate the samples despite the near anoxic groundwater environment (Table 3 and Figures 3, 4). Signatures of microbes representing aerobic methanotrophic metabolisms were detected in fluids from the deepest and shallowest wells of CROMO (QV1-3, 35.2 m and CSW1-4, 8.8 m respectively). Similar evidence was seen in samples from QV1-2 (at ~14 m depth) and from biofilm swabbed from suspended equipment in the uncased, deep well (CSW1-1, at ~19 m depth). Differences in methane emissions were noted after we pumped standing water (Table 4) and we found that recharged groundwater in wells demonstrated a higher proportion of methanotrophic signatures than the standing water.

Lipid signatures for bacteria were in every one of the seven wells sampled. Partial indicators of aerobic methanotrophy were found in six wells (Supplementary Table ST1) and definitively indicated in four wells, based on the two sequential patterns of monounsaturated 16:1 and 18:1 fatty acids. Type I (gammaproteobacterial) methanotrophs were more prevalent in our shallow well, CSW1-4, and Type II (alphaproteobacterial) were more prevalent in the deeper well (QV1-3). This is consistent with laboratory experiments, in which Type I aerobic methanotrophs outcompete Type II in lower methane/higher oxygen environments, while Type II are favored in high methane environments (Hanson and Hanson, 1996). Biofilm from the ~19-m-deep, uncased well (CSW1-1, ultrabasic pH > 12) also had a higher proportion of Type II methanotrophs (Table 5).

**Table 5:** The abundance of aerobic methanotrophs was assessed semiquantitatively by quantifying the concentration of the monounsaturated fatty acids in the cells and normalizing them to the dominant membrane fatty acid.

Sample	H20 (mL)	Type I	Type II	Type I: Type II
		16:1/16:0	18:1/18:0	16:1/18:1
CSW1-1_biofilm	N/A	0.1	1.1	0.1
CSW1-4_DR	437	2.4	4.9	2.8
CSW1-4_MR	696	5.3	12.8	2.6
QV1-3_DR	157	1.6	3	0.5

D = December, M = May, S = Standing, R = recharged. Below represents one filter per well, rather than combined samples in Figure 3. Type I are Gammaproteobacterial methanotrophs and Type II are Alphaproteobacterial methanotrophs. Highlighted text (orange used for CSW1-4 throughout, blue for QV1-3) signifies that DMDS adduction was completed to verify carbon bond positions of unsaturated peaks. The monounsaturate16:1/18:1 ratio, that estimates the Type I to Type II types, is based on relative values of the monounsaturates alone, as listed in Supplemental Table ST1.

Differences in methane emissions were noted after we pumped standing water out (Table 4) and we found that recharged groundwater in wells demonstrated a higher proportion of methanotrophic signatures than the standing water, along with differences in geochemistry (Table 3). Microbial detection in lipid samples contrasts with earlier genetic analysis at CROMO (Crespo-Medina et al., 2014; Twing et al., 2017; Sabuda et al., 2021), which showed an abundance of signatures from Betaproteobacteria, Deinococci and/or orders and classes within Bacillota. Differences in sampling may have had some impact. However, Type I methanotrophs were clearly identified in Seyler et al. (2020) within CSW1-1 and QV1-1, and aerobic methane oxidation was suggested in metagenomic and metatranscriptomic data in all wells they sampled, with ‘sparse’ indicators of Type II metabolism. We discovered lipid signals for Type I and Type II methane oxidizers in QV1-2; QV1-3 was not sampled in either previous study. These other phylotypes were likely to be present in our samples, as they have also been detected in serpentine-influenced aquifers at Tablelands, The Cedars and Cabeço de Vide (Brazelton et al., 2012; Suzuki et al., 2013; Tiago and Veríssimo, 2013), but here their biomarkers were not comparable in strength. CSW1-1 biofilm from standing water had a relatively diverse range of lipid signals, inviting future scrutiny (Supplementary Table ST1).

An established advantage of lipid analysis is that it directly measures the presence or absence of intact membranes, capturing intact phospholipids before these molecules degrade to free fatty acids upon cell death (White et al., 1993; Parenteau et al., 2014). The dominance of methanotrophs emerges from semi-quantification possible with FAMEs. This relative quantification of lipids may be sensitive to the relative size of methanotrophic cells. Reis et al. (2022) noted that, due to large cell size, aerobic methanotrophs in a low-oxygen aqueous ecosystem made up 1.3% of a cell count but 17% of biomass. FAME analysis may be comparable to measurements of labile mRNA molecules reverse-transcribed to cDNA, which suggest activity of live cells. In a Samail Ophiolite study by Kraus et al. (2021), Type I methanotrophs comprised a greater percentage of the cDNA of a total population (representing metabolically active cells) compared with the DNA. Seyler et al. (2020) also noted a higher proportion of methanotrophs in metatranscriptomes than in metagenome data at CROMO. Our work may agree with Reis et al.’s (2022) suggestion that these cells have a more significant metabolic contribution to a microbial community than cell or gene counts may imply. Sensitivity of lipid analyses is appropriate to low biomass noted in CROMO groundwater (Crespo-Medina et al., 2014) and allows estimates of cells. We estimate a sample with some of the most robust signals as 1.01 × 10^6^ cells per mL (Supplementary Table ST2), consistent with previous estimates of 10^5^ to 10^6^ cells/mL (Twing et al., 2017) and otherwise observed in serpentinizing fluids (e.g., Meyer-Dombard and Malas, 2022).

These methods may be expanded upon for other lipid biomarkers. The rare stability among biological molecules have long recommended lipids to inquiry into the origin and presence of life, as a biosignature in both early Earth and planetary research (Sánchez-García et al., 2023). We were able to identify specific fatty acids with about 1/20 the minimum water volume used in previous molecular work (Supplementary Table ST1). A method to further concentrate a lipid signal could continue to develop these data, given very slow groundwater flow rates and large geologic unit volumes, which cause a low yield of water. Pooling biomass would expand detection to other molecules of interest to questions of biosignatures, deep time and methanotrophy, such as hopanoids (Jahnke et al., 1992), sterol-like lipids found in a lipid survey of Samail ophiolite rock cores (Newman et al., 2020). Analysis of fatty acid methylation could also be used as well. Along with detection of NC10 bacteria (Kool et al., 2012), methylation may be used to evaluate responses to environmental conditions and community dynamics. These techniques can all be used in benchtop experiments analogous to low-oxygen, high-pH sites and the specific geochemistry described here, while in the field, more frequent sampling could reveal seasonal trends in biotic carbon cycling, indicated in our work by differences between May (dry season) and December (rainy season) measurements (Supplementary Table ST1).

### 4.2 Limitations of lipid signals diagnostic for other microbial types

We attempted to identify methanogens and ANME by archaeol (Jahnke et al., 2008) in our nine TMS-adducted MAH samples. However, archaeol, universal to Archaea, was not detected (Supplementary Figure SFI), and therefore both methanogens and ANME populations are at levels below detection in our samples, if present. We targeted sulfate-reducing bacteria as well to indicate contextual support for ANME, as SRB presence can suggest ANME due to a syntrophic relationship. While some features of SRBs were found in low levels of terminally branched iso and anteiso fatty acids (Figure 3), SRBs may also be marked by DAGE/MAGE (Parenteau et al., 2014), which we did not detect (Supplementary Table ST1). Since SRBs have been previously detected at CROMO (Sabuda et al., 2021), it is worth future work to target the lipid signatures pertinent to this group.

Lipid biosignatures for other microbes are present but weaker in signal than the series of monounsaturates. The alkaliphile strain of *Serpentinimonas* is similar to a Betaproteobacterial family detected at CROMO (Twing et al., 2017; Seyler et al., 2020). Its consistent, dominant fatty acids are C_15_ (Thompson et al., 2023), rather than the C_16:1_ signatures, although they generally contain one C_16:1_ peak (Bird et al., 2021; Thompson et al., 2023). Our samples contained isomers of C_15_ (Figure 3) but at levels far below the 40% relative abundance in *Serpentinimonas* (Bird et al., 2021). Members of a phylum previously found at CROMO, Bacillota (Firmicutes) (Twing et al., 2017), may contain C_16:1_ isomers in low levels (Fulco, 1967). C_14_ and C_15_ are dominant in Bacillota (Elsden et al., 1980). *Dethiobacter*, found at CROMO (Putman et al., 2021) has high levels of C_14_ (Sorokin et al., 2008), and C_14_ can compose up to 47% of fatty acids in the *Clostridium* genus (Moss and Lewis, 1967), dissimilar to our samples. In the mixed community we found approximately 5% relative abundance of C_14_, also produced by methanotrophs but low compared to the major three 16:1 signals that were 17 to 52% of the fatty acid pool. Finally, a member of the Deinococcota phylum at CROMO, *Truepera* (Sabuda et al., 2021) displays C_anteiso-15:0_, C_iso-17:0_, and C_iso-16:0_ (Ivanova et al., 2011), which are present but underrepresented in our samples. The novel data in this study suggest that it is not these bacteria but methanotrophs that explain the dominant patterns in the lipids.

### 4.3 Abiotic origin of methane at CROMO supported by lipid investigation, ^14^C radioisotope, and δ^13^C data

Modern methane is likely to be generated by either biogenesis or recent geogenesis, while ancient methane can have geogenic or thermogenic origin. In our data, methanogens are below detection levels in groundwater and aqueous radioisotope data did not support recent, biotic methanogenesis, which could be a negligible source of methane at CROMO.

Methanogens have recently been detected in rock material from the CROMO area but not in any groundwater samples to date (Twing et al., 2025) and their significance in output of methane remains unclear. While we demonstrated our ability to find archaeol, a biomarker for methanogens and ANME in samples (Supplementary Figure SF1), none was located in nine samples taken from groundwater in wells CSW1-1, CSW1-4, CSW1-5, and QV1-3 (Supplementary Table ST1).

Radiocarbon data suggests very little modern methane (the speculative biogenic fraction due to modern methanogenesis): 4% in CSW1-4 and 0.08% in QV1-3. Soil typically has a radiocarbon age of 4,830 ± 1,730 yr. (Shi et al., 2020), which is younger than the methane in groundwater of shallow, soil-influenced CSW1-4, indicating that its groundwater draws on older methane.

Methane from the deepest well appears to be ancient in origin, but carbon in DIC samples is more recent (Table 2). ^14^C of methane for our deepest well, QV1-3, was ancient (here, reported as >48,000), with younger DIC (9,500 y BP). ^14^C analyses from the Ronda peridotite massif in southern Spain show similar results, with ancient methane and DIC in the thousands of years BP (Etiope et al., 2016); Suda et al. (2022) also found ancient methane in a hyperalkaline spring in Hakuba Happo, Japan, using carbonate precipitates as a constraint. Methane may therefore be generated from ancient carbon rather than recent carbon in the atmosphere. A (relatively lesser) depletion in carbon of DIC could be from methanotrophy or from progressive carbonate precipitation (Ternieten et al., 2021). Stable isotope (δ^13^C) data (Table 2) also may indicate biotic or abiotic redox reactions (Kietäväinen and Purkamo, 2015; Milkov and Etiope, 2018). Following Whiticar’s (1999) plot of methane vs. carbon dioxide (part of the DIC) in water, QV1-3 is nearest the range of the methane oxidation zone and CSW1-4 is firmly in this zone. In future work, this data set could be expanded to include ^13^C analysis of cell membranes through compound-specific isotope analysis. Using clumped isotopes (Δ^13^CH_3_D), which indicate the temperature at which C-H bonds were formed, Wang et al. (2015) suggested that methane in the McLaughlin Natural Reserve (sampled from older mining wells pre-dating CROMO) could indicate geogenic Sabatier/FFT-resulting hydrogen sources. However, with low levels of dissolved hydrogen with respect to other continental systems (Crespo-Medina et al., 2014), conditions for methanogenesis at CROMO are underwhelming. Our data indicate that microbial methanogenesis is not a major source of methane at CROMO, supporting earlier observations, and suggest that methanotrophy may actually be a source of inorganic carbon in the groundwater.

### 4.4 Geochemistry of groundwater, methane emissions, and support for microbial communities

On the whole, aqueous geochemical data show differences in solute and ion levels in standing and recharged waters. This finding, combined with documentation of shifting methane emission levels, is likely to influence microbial populations. Since only standing waters were sampled prior to this work, microbial ecosystem dynamics deserve a continued and more detailed description to characterize the recharged waters, fresh from the formation.

In geochemical features of actively serpentinizing systems, open system (in contact with modern atmosphere) and closed system (deeply sourced, isolated from atmosphere) waters have different major ion characteristics.

**Figure 5:**
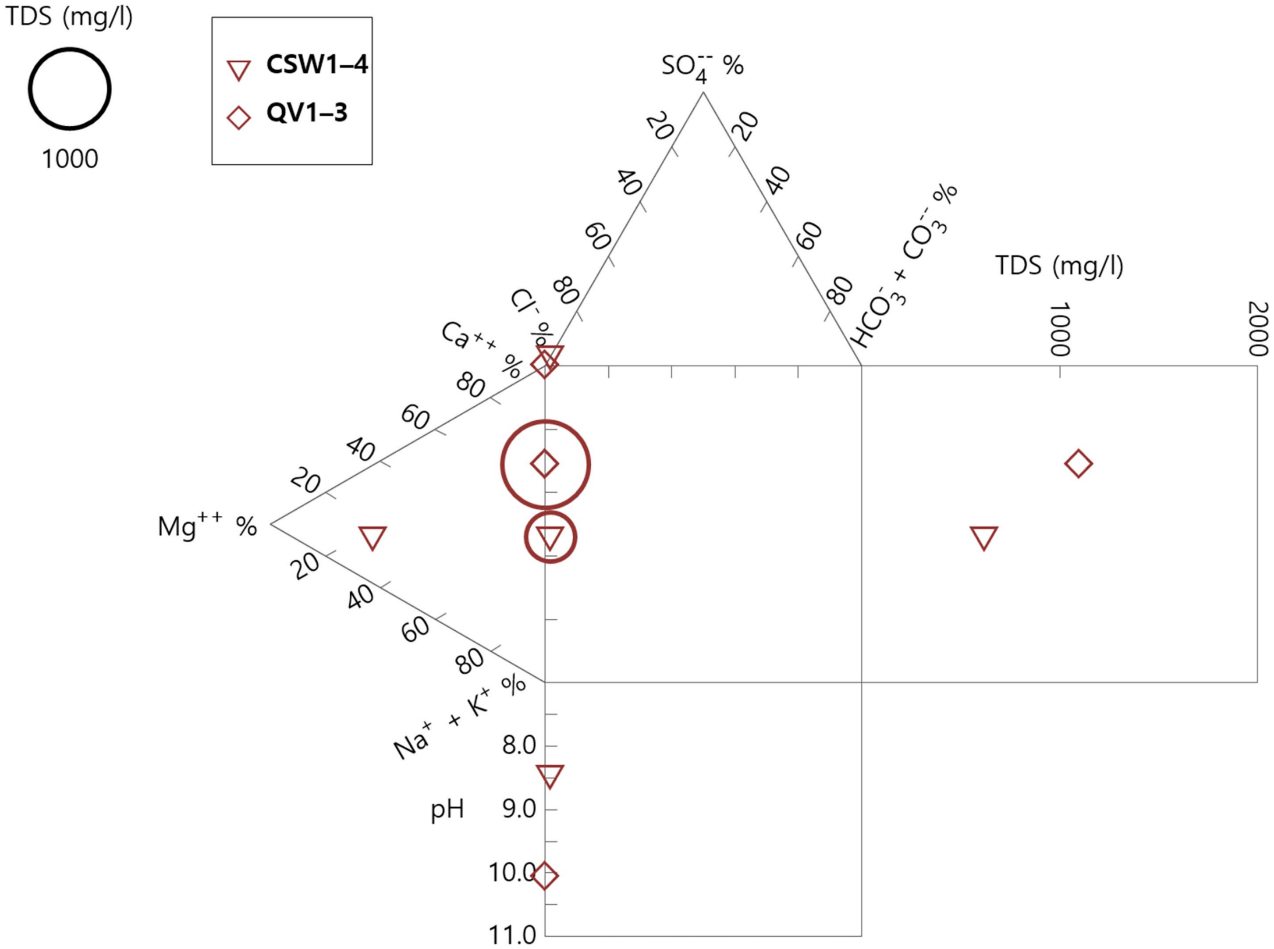
Characteristics of groundwater at CROMO represent Type I (Mg^2+^-HCO_3_^−^) and Type II waters (Ca^2+^-OH^−^) for the deepest (QV1-3) and shallowest (CSW1-4) wells sampled here. The Durov diagram displays higher Mg^2+^ and moderate pH in CSW1-4 and higher Ca^2+^- and pH in QV1-3, indicating Type I and Type II, respectively. Total dissolved solids (TDS) are represented. Higher loads in QV1-3 may reflect the more concentrated biomass found in the lipid sample.

Bearing this in mind, we noted a dominance of serine-based metabolisms in Type II (older) waters and RuMP-based in Type I (younger) waters. Recall the two major water types in serpentine-influenced groundwater are Mg^2+^-HCO_3_^−^ (Type I waters) vs. Ca^2+^-OH^−^ (Type II waters), respectively, with characteristic differences in Ca/Mg ratio and pH (Neal and Stanger, 1985; Morrill et al., 2013). The Type II waters may be at least 20,000 years old for some sites (Vankeuren et al., 2019). The ions predominant in wells correlated with ancient Type II waters (QV1-3) and a mixture of young Type I and ancient waters (CSW1-4). The most surface-influenced water (CSW1-4) had higher levels of magnesium than deeper wells, indicating open system water infiltration, while the deeper QV1-3 had relatively high pH and calcium, indicating closed system water infiltration and characteristic of subsurface serpentinization (Barnes et al., 1967) and/or the *in situ* chemical weathering of serpentinites (McCollom and Shock, 1997) (Figure 5). The well with greater Type I influence (CSW1-4) also had more prominent differences in ORP and some ion levels relative to other wells (Supplementary Table ST4). Ophiolite-hosted waters, with their long-term stability (Colman et al., 2025), have been shown to promote speciation of microbes (Munro-Ehrlich et al., 2023). Intriguing work in the Beatrix Gold Mine, South Africa, has proposed a paradigm of electron availability (fundamentally tied to shifts in aqueous geochemistry) impacting methane oxidizers (Magnabosco et al., 2018b). Further investigation has yet to be done on water-type support of specific microbes at a serpentinization-influenced site.

At CROMO, hydrogen emissions, a major source of energy for methanogens, are low in comparison to other ophiolites and hydrothermal sites (Sabuda et al., 2021; Twing et al., 2017). This may be a result of the CROMO system’s state of advanced alteration, with more than 70% serpentine minerals making up the ultramafic country rock with “minor silica carbonate rock,” indicating late stage SiO_2_- and CO_3_^2−^-bearing waters active in the landscape, observed (Williams, 1984).

Dissolved oxygen (DO) diffuses into the water from above, where the well pipes open to the atmosphere, but rapidly falls to near zero with depth, as shown by incrementally submerging a fiber optic DO probe cable from the well top. DO approached zero at 2.4 m for CSW1-1 and 3.9 m for QV1-3 (Figure 4), corresponding with previous year-round (but less sensitive) YSI meter measurements. YSI-measured DO approached zero at 5.9 m below land surface at CSW1-1 (Sabuda et al., 2021). In measurements in the past for the wells of interest, as reported in Table 3 and in Supplementary Table ST4, the DO has been recorded by YSI as low as 0.3 mg/L in QV1-3 (Seyler et al., 2020). These observations, coupled with this study’s findings that standing vs. recharged oxygen levels differ, call for further study. Certainly, this brings questions about the presence of aerobic methanotrophs in water recharged from bedrock, especially where recharged waters appear to have different characteristics than standing water (Supplementary Table ST1) despite the influence of atmospheric DO.

We measured nitrate in the standing water of three wells (Table 3), which may be used by Type I methanotrophs in hypoxic conditions (Guerrero-Cruz et al., 2021). We found pyruvate in QV1-3, an intermediate in Type I and II methanotrophs (Hanson and Hanson, 1996). Nutrients and byproducts of other microbes, such as acetate and butyrate, were below detection in recharged CSW1-1 water although previously reported in its standing water (Twing et al., 2017; Seyler et al., 2020) using different analytical techniques. Certain cations (copper, iron and others) stabilize methane monooxygenase (MMO), but we did not have detectable copper in wells. Iron was only detectable in CSW1-1. This may be because methanotrophs scavenge and sequester copper, or use ions that include silver, gold, iron, nickel, cobalt and potassium (Dershwitz et al., 2021).

Recent recharge of water appears to affect methane concentration levels and diversity of biological signals (Tables 4, 5 and Figure 4). Recharged fluids generally had a much higher methane contents than before purging wells. Explanations for the pre- and post-purge differences in methane concentrations detected at the wellheads may include: (1) the standing column of water in the pre-purge condition could physically act as a cap (or “plug”) for the methane, holding it in the dissolved phase; (2) the loss of the standing column of water in the post-purge condition could allow a subsurface, fracture-controlled methane seep free egress; (3) methanotrophs present in large abundance in the standing column of water in the pre-purge (stable for periods of months to years) condition could rapidly utilize methane, serving as a biological filter (Hanson and Hanson, 1996; La et al., 2018), preventing methane escape.

More generally, variability in methane emissions at the CROMO wells, which were here used as a proxy for dissolved methane, must affect methanotrophs and therefore total biomass. Their activity could be interrupted, given the literal upset of recharging waters flowing into the well at its bottom (i.e., through fine holes/slits cut into the well’s screened pipe section). In the shallowest well, CSW1-4, emissions were lowered to atmospheric levels again upon recharge of the ‘new’ waters. However, all other wells, with ‘older’ water, displayed methane emission levels of several orders of magnitude higher in tandem with recharged waters and diminished after recharge in four out of six wells (Table 4 and Supplementary Figure SF2). The high variability that we detected correlates with previous ophiolite and subsurface surveys. Etiope et al. (2016) reported concentrations that ranged from 4 to 230 ppm, and flux at 5 to 464 mgm^−2^day^−1^ at a seep, and Leong et al. (2023) reports outgassing from seeps as 640 mol/yr. from a diffuse area of a site to 6,700 mol/yr. to a bubbling area (Table 4).

We link methane emissions, subsurface water chemistry and biomass due to influx of waters and seasonal changes in biomass concentration for select wells. Differences in emissions and aqueous chemistry, and therefore the bioenergetics, could be linked to the effect of seasonality on biomass in future work. Previous work at CROMO that identified the groundwater stand/perched aquifer dimensions and dynamics noted seasonality of water characteristics in terms of resistivity and ORP data (Ortiz et al., 2018). A relationship with seasonal differences in dissolved methane and oxygen was also noted at CROMO (Crespo-Medina et al., 2014) and could be further linked to biomass and even competition between Type I and Type II methanotrophs for resources – namely, methane and nitrate (Graham et al., 1993; Jahnke et al., 1999). With documentation of changes in emissions and chemistry in relation to the movement and presence of subsurface water and our profiling of methanotrophs, closer study of the connections between subsurface site conditions and near-surface methane emissions is warranted.

### 4.5 Conclusion and outlook

This work underscores the significance of aerobic methanotrophy in a serpentinization-influenced groundwater system. We report lipid-based detection of both major aerobic methanotrophic metabolisms within the microbial communities at CROMO and across multiple wells with differing local aquifer conditions.

Identification of methanotrophs and an absence of methanogens is cross-referenced here with radiocarbon and stable isotope data for methane and DIC, along with geochemistry of the groundwater. The radiocarbon data indicate ancient, abiotic methane but younger DIC, potentially an output of methanotrophs. Together, data suggest a biological sink of methane, and imply that methane emissions at CROMO may be primarily geogenic/thermogenic in origin.

Our data emphasize the importance of water conditions to biotic carbon cycling in serpentinite-hosted waters. We show a dominance of Type II methanotrophs in recharged, closed-system Ca_2+_-OH- type water (well QV1-3), and find that Type I (Gammaproteobacteria) dominate open-system Mg_2+_-HCO_3_- type water in a shallower well (CSW1-4, anchored in serpentine-rich soil). Our results also correspond with studies that monitored and detected aerobic methanotrophs in conditions of low oxygen (Jahnke and Nichols, 1986; Reis et al., 2024) and ^14^C-depleted groundwaters (Ruff et al., 2023).

Despite the challenges of high pH and low oxygen at CROMO, methanotrophic communities play an underdescribed role in subsurface rock-hosted microbial ecosystems in this site. This finding begs renewed investigation of methanotrophy at related sites. Given the importance of the continental subsurface to the total biomass on Earth (Magnabosco et al., 2018a), the role of methanotrophs in these serpentinization-influenced systems needs to be more closely defined. Their significance as purveyors of change in the global carbon cycle has likely been underestimated.

## Data Availability

The original contributions presented in the study are included in the article/Supplementary material, further inquiries can be directed to the corresponding author.

## Footnotes

1
https://www.geotechenv.com/geotech_bladder_pumps.html

2
http://licor.com

3
https://www2.whoi.edu/site/nosams/
