# Supplementary material for: Lipid biomarkers reveal dominance of aerobic methanotrophy in a continental serpentinizing system
Source: Front Microbiol. 2026 Mar 5;16:1694997. doi: 10.3389/fmicb.2025.1694997 (PMC13001230; doi:10.3389/fmicb.2025.1694997)
Supplement: Supplementary file 1 [file Supplementary_file_1.docx]

**
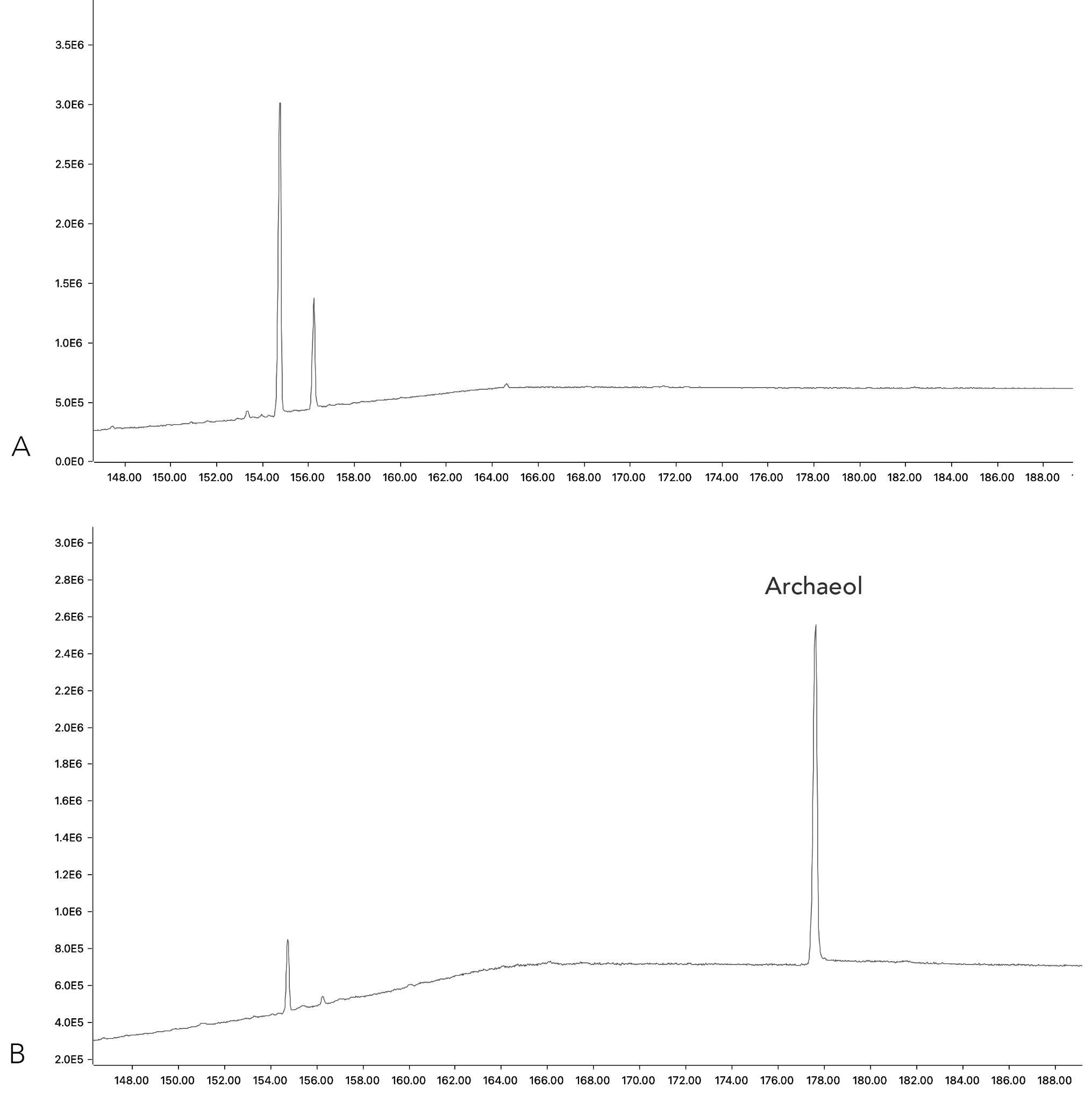
**

**SF1 (Supplementary Figure 1): Absence of archaeol shown, the ubiquitous biomarker found in methanogens and methanotrophs. Archaeol may be found on the biocontrol (B) at 177.8 minutes elution time, and is absent in well samples. The sample at top (A), QV1-3 represents eight other attempts.**
