# Supplementary material for: Lipid biomarkers reveal dominance of aerobic methanotrophy in a continental serpentinizing system
Source: Front Microbiol. 2026 Mar 5;16:1694997. doi: 10.3389/fmicb.2025.1694997 (PMC13001230; doi:10.3389/fmicb.2025.1694997)
Supplement: Supplementary file 2 [file Supplementary_file_2.docx]

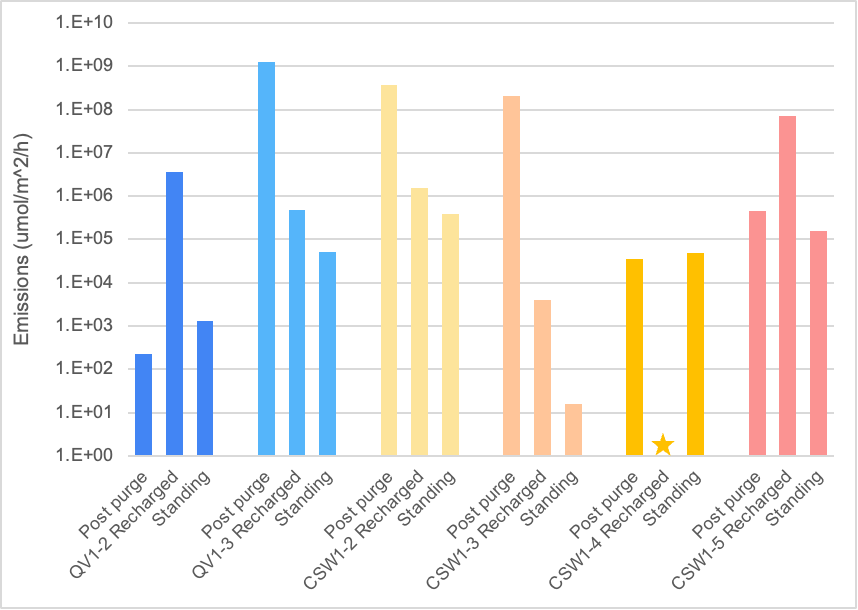


**SF2 (Supplementary Figure 2): Emissions from wells represented as estimated flux for all 2-inch wells: QV1-2, QV1-3, CSW1-2, CSW1-3, CSW1-4, CSW1-5. Plotted in log value, so the negative flux of CSW1-4 (recharged) is not graphed. Standing water represents four months of inactivity, so here we report it after recharged wells, instead of chronologically. All measurements shown here were measured with one input, but dual inputs (to attempt to capture heterogeneity of methane emissions within the well space) were used to measure for standing water, and the QV1-2 standing measurement had a dual input (single was not recorded).**
