# Supplementary material for: Lipid biomarkers reveal dominance of aerobic methanotrophy in a continental serpentinizing system
Source: Front Microbiol. 2026 Mar 5;16:1694997. doi: 10.3389/fmicb.2025.1694997 (PMC13001230; doi:10.3389/fmicb.2025.1694997)
Supplement: Supplementary file 5 [file Table_3.docx]

**ST3 (Supplementary Table 3). Expanded set of stable and radioisotope data taken in May 2024 from recharged waters of four wells.**

|  | **CSW1-3** | **CSW1-4** | **CSW1-5** | **QV1-3** |
| --- | --- | --- | --- | --- |
| ***δ^13^C*** |  |  |  |  |
| DIC (permil) | -3.9 | -18.64 | -7.93 | -33.79 |
| CH_4_ (permil) | -26.38 | -31.05 | N/A | -26.25 |
| ***^14^C*** |  |  |  |  |
| DIC F Modern | 0.2627 +/- 0.0049 | 0.5073 +/- 0.0015 | 0.5724 +/-0.0039 | 0.3065 +/- 0.003 |
| DIC Age | 10750 +/- 150 | 5450 +/- 25 | 4480 +/- 55 | 9500 +/- 80 |
| ***^14^C*** |  |  |  |  |
| CH_4_ F Modern | 0.0044+/- 0.0016 | 0.0366 +/- 0.0022 | N/A | 0.0008 +/- 0.0016 |
| CH_4_ Age | 43500 +/- 2800 | 26600 +/- 490 | N/A | > 48000 |
