# Supplementary material for: Lipid biomarkers reveal dominance of aerobic methanotrophy in a continental serpentinizing system
Source: Front Microbiol. 2026 Mar 5;16:1694997. doi: 10.3389/fmicb.2025.1694997 (PMC13001230; doi:10.3389/fmicb.2025.1694997)
Supplement: Supplementary file 6 [file Table_4.docx]

**ST4 (Supplementary Table 4). Environmental parameters observed in past publications at the Core Shed and Quarry Valley wells**.

|  | **CSW1.1** | **CSW1.2** | **CSW1.3** | **CSW1.4** | **CSW1.5** | CSWold | **QV1.1** | **QV1.2** | **N08A** | **N08B** | **N08C** |
| --- | --- | --- | --- | --- | --- | --- | --- | --- | --- | --- | --- |
| **Temperature (C)** |  |  |  |  |  |  |  |  |  |  |  |
| Twing 2017 | 17.2 | 18.5 | 16.9 | 15.2 | 16.2 |  | 17.9 | 18.4 |  |  |  |
| Seyler 2020 | 16.1 |  | 16.662 | 13.81 |  | 17.45 ± 0.45 | 15.667 ± 0.677 | 16.647 ± 0.63 | 16.16 | 15.7 | 15.65 |
| Sabuda 2021 | 18.7 |  |  |  |  |  |  |  |  |  |  |
| Crespo-Medina 2014 March | 14.9 |  |  |  |  | 17.7 |  |  |  | 14.7 | 14.6 |
| Crespo-Medina 2014 August | 16.2 |  |  |  |  | 18.2 |  |  |  | 16 | 15 |
| **pH** |  |  |  |  |  |  |  |  |  |  |  |
| Twing 2017 | 12.2 | 9.3 | 10.1 | 7.9 | 9.7 |  | 11.5 | 7.9 |  |  |  |
| Seyler 2020 | 12.345 ± 0.195 |  | 10.12 | 7.77 |  | 9.765 ± 0.075 | 11.478 ± 0.083 | 8.663 ± 0.367 | 10.535 ± 0.015 | 10.942 ± 0.158 | 7.89 |
| Sabuda 2021 | 12 |  |  |  |  |  |  |  |  |  |  |
| Crespo-Medina 2014 March | 12.2 |  |  |  |  | 9.8 |  |  |  | 10.7 | 7.4 |
| Crespo-Medina 2014 August | 12.4 |  |  |  |  | 9.8 |  |  |  | 11 | 7.6 |
| **ORP (mV)** |  |  |  |  |  |  |  |  |  |  |  |
| Twing 2017 | -284 | −32 | −83 | −35 | -121 |  | −155 | −30 |  |  |  |
| Seyler 2020 | -195.8 |  | -97.3 | -98.2 |  | -346 | -168.233 ± 40.383 | -54.8 ± 34.82 | -69.7 | -69.46 ± 24.884 | -25.4 |
| Sabuda 2021 | -262 |  |  |  |  |  |  |  |  |  |  |
| Crespo-Medina 2014 March | −292.8 |  |  |  |  | −220.4 |  |  |  | −126.5 | −127.6 |
| Crespo-Medina 2014 August | -254.9 |  |  |  |  | −277.5 |  |  |  | −65.3 | 217.3 |
| **DO (mg/L)** |  |  |  |  |  |  |  |  |  |  |  |
| Twing 2017 | 0.05 | 0.41 | 0.06 | 1.05 | 0.03 |  | 0.03 | 0.03 |  |  |  |
| Seyler 2020 | 0.64 |  | 0.07 | 0.86 |  | 0.22 | 0.392 ± 0.214 | 0.427 ± 0.206 | 0.17 | 0.214 ± 0.069 | 0.11 |
| Crespo-Medina 2014 March | 0.5 |  |  |  |  | 8.8 |  |  |  | 0.38 | 3.12 |
| Crespo-Medina 2014 August | 0.2 |  |  |  |  | 0.02 |  |  |  | 0.31 | 0.17 |
| **Conductivity (µS/cm)** |  |  |  |  |  |  |  |  |  |  |  |
| Twing 2017 | 5200 | 3710 | 4500 | 1560 | 4220 |  | 2068 | 1655 |  |  |  |
| Seyler 2020 | 5.069 |  |  |  |  |  |  |  |  |  |  |
| Sabuda 2021 | 3820 |  |  |  |  |  |  |  |  |  |  |
| Crespo-Medina 2014 March | 4.5 |  |  |  |  | 8.2 |  |  |  | 2.9 | 0.8 |
| Crespo-Medina 2014 August | 4.5 |  |  |  |  | 11.2 |  |  |  | 0.31 | 0.17 |
| **DOC (µM)** |  |  |  |  |  |  |  |  |  |  |  |
| Crespo-Medina 2014 March | 1163 |  |  |  |  | 27 |  |  |  | 67 | 408 |
| Crespo-Medina 2014 August | 989 |  |  |  |  | 93 |  |  |  | 21 | 1144 |
| **DIC (µM)** |  |  |  |  |  |  |  |  |  |  |  |
| Twing 2017 | 253 ±8 | 605 ±268 | 172 ±16 | 5046 ±531 | 545 ±13 |  | 96 ±2 | 979 ±32 |  |  |  |
| Seyler 2020 | 149.91 ± 103.51 |  | 171.89 | 2494.6 |  | NA | 48.185 ± 12.699 | 827.5 ± 151.93 | 63.435 ± 7.485 | 40.948 ± 8.788 | 1023.4 |
| Sabuda 2021 | 655 |  |  |  |  |  |  |  |  |  |  |
| Crespo-Medina 2014 March | 210 |  |  |  |  | 63 |  |  |  | 22 | 1831 |
| Crespo-Medina 2014 August | 194 |  |  |  |  | 42 |  |  |  | 98 | <0.003 |
| **Dissolved H2 (µM)** |  |  |  |  |  |  |  |  |  |  |  |
| Twing 2017 | 0.289 ±0.004 | 0.140 ±0.001 | 0.283 ±0.018 | 0.271 ±0.013 | 0.138 ±0.020 0.075 ±0.001 0.076 ±0.009 |  | 0.075 ±0.001 0.076 ±0.009 | 0.076 ±0.009 |  |  |  |
| Seyler 2020 | 0.2887 |  | 0.2837 | 0.7505 |  | NA | 0.255 ± 0.169 | 0.109 ± 0.033 | 0.103 ± 0.095 | 0.194 ± 0.102 | 0.724 |
| Sabuda 2021 | 0.02 |  |  |  |  |  |  |  |  |  |  |
| Crespo-Medina 2014 March | 0.0128 |  |  |  |  | <0.003 |  |  |  | 0.1501 | <0.003 |
| Crespo-Medina 2014 August | 1.4822 |  |  |  |  | <0.003 |  |  |  | 3.1 | 1.1 |
| **Dissolved CH4 (mM)** |  |  |  |  |  |  |  |  |  |  |  |
| Twing 2017 | 0.524 ±0.132 | 1.625 ±0.055 | 0.969 ±0.529 | 0.002 ±0.0003 | 1.266 ±0.032 |  | 0.301 ±0.021 | 0.303 ±0.030 |  |  |  |
| Seyler 2020 | 0.5235 |  | 0.9694 | 0.0034 |  | NA | 0.301 ± 0.022 | 0.319 ± 0.016 | 1.184 ± 0.206 | 0.471 ± 0.123 | 0.004 |
| Sabuda 2021 | 0.452 |  |  |  |  |  |  |  |  |  |  |
| Crespo-Medina 2014 March | 0.455 |  |  |  |  | 0.680 |  |  |  | 0.338 | <0.003 |
| Crespo-Medina 2014 August | 0.613 |  |  |  |  | 0.1983 |  |  |  | <0.003 | 0.017 |
